# Discrete Element Framework for Modelling Extracellular Matrix, Deformable Cells and Subcellular Components

**DOI:** 10.1371/journal.pcbi.1004544

**Published:** 2015-10-09

**Authors:** Bruce S. Gardiner, Kelvin K. L. Wong, Grand R. Joldes, Addison J. Rich, Chin Wee Tan, Antony W. Burgess, David W. Smith

**Affiliations:** 1 School of Engineering and Information Technology, Murdoch University, Perth, Australia; 2 Engineering Computational Biology, School of Computer Science and Software Engineering, The University of Western Australia, Perth, Australia; 3 Intelligent Systems for Medicine Laboratory, School of Mechanical and Chemical Engineering, The University of Western Australia, Perth, Australia; 4 Structural Biology Division, The Walter and Eliza Hall Institute of Medical Research, Parkville, Victoria, Australia; 5 Department of Medical Biology, University of Melbourne, Melbourne, Victoria, Australia; 6 Department of Surgery, Royal Melbourne Hospital, Melbourne, Victoria, Australia; University of Virginia, UNITED STATES

## Abstract

This paper presents a framework for modelling biological tissues based on discrete particles. Cell components (e.g. cell membranes, cell cytoskeleton, cell nucleus) and extracellular matrix (e.g. collagen) are represented using collections of particles. Simple particle to particle interaction laws are used to simulate and control complex physical interaction types (e.g. cell-cell adhesion via cadherins, integrin basement membrane attachment, cytoskeletal mechanical properties). Particles may be given the capacity to change their properties and behaviours in response to changes in the cellular microenvironment (e.g., in response to cell-cell signalling or mechanical loadings). Each particle is in effect an ‘agent’, meaning that the agent can sense local environmental information and respond according to pre-determined or stochastic events. The behaviour of the proposed framework is exemplified through several biological problems of ongoing interest. These examples illustrate how the modelling framework allows enormous flexibility for representing the mechanical behaviour of different tissues, and we argue this is a more intuitive approach than perhaps offered by traditional continuum methods. Because of this flexibility, we believe the discrete modelling framework provides an avenue for biologists and bioengineers to explore the behaviour of tissue systems in a computational laboratory.

## Author Summary

Modelling is an important tool in understanding the behaviour of biological tissues. In this paper we advocate a new modelling framework in which cells and tissues are represented by a collection of particles with associated properties. The particles interact with each other and can change their behaviour in response to changes in their environment. We demonstrate how the propose framework can be used to represent the mechanical behaviour of different tissues with much greater flexibility as compared to traditional continuum based methods.

## Introduction

The quality and scope of experimental data on cells and tissues has undergone rapid advances. High throughput technologies have given unprecedented insight into signal transduction, gene activation, and associated cell decision processes. New techniques have also enabled the physical manipulation of cells, which has spurred the potential for deeper understanding of cell-cell and cell-ECM (extracellular matrix) physical interactions [1]. Taken together, there is an opportunity to integrate this information into computational models that are capable of representing both the mechanical and chemical interactions in biological systems. The modelling frameworks that are most appropriate for the new types of problems and data sets presented by biological systems are yet to be determined.

Tissues are generally in a state of flux. That is, an apparently static tissue is actually maintaining itself through continual renewal. Cells maintain themselves, proliferate, grow, differentiate, secrete and migrate to new locations, often undergoing substantial morphological change during these processes. The extracellular matrix is also continually ‘turned over’ and/or remodelled. It is therefore highly desirable to have a modelling environment that can easily represent very large deformations and other morphological changes in cells and the extracellular matrix, along with physical interactions between cells and cells and the extracellular matrix. It is also now apparent that cells behave as wet 'computers' for processing environmental information and forming appropriate responses to environmental signals. It is therefore highly desirable to accommodate decision logics in the modelling environment, based on the internal state of the cell and its external environment.

Traditional modelling approaches have usually relied upon continuum mechanics modelling based on finite element or finite difference representations of partial differential equations [2–5]. The continuum approaches rely upon ‘homogenisation’ techniques, which by design average out lower scale information. This reduces the complexity of the model, but when the complexity of the lower scale has a strong influence at the scale of the problem, the complexity returns in the form of a complex constitutive law. This approach has been very useful in understanding the load-deformation of hard tissues such as bone, and some soft tissues such as cartilage [6,7]. However, these models need to pre-define a problem domain and can only model events requiring evolution of the spatial domain of interest with considerable difficulty (e.g. growth, fractures, contacts, multiphase processes). Typically the continuum mechanics models are based on advanced mathematical concepts and produce outputs that are often abstract representations of what a biologist observes through a microscope, so this type of modelling output is often non-intuitive to biologists and they struggle to engage with the methodology (which in unsurprising given that it usually takes engineers and mathematicians years to master the techniques).

To overcome some of the limitations of continuum mechanics modelling, lower scale continuum models could be developed and coupled together to form multiscale models, to better represent heterogeneous tissues. In a biological context, one faces the problem of developing multiscale methods that can model extremely large deformations coupled with cell proliferation, growth and migration (often with changes in material properties); this presents enormous numerical challenges for conventional continuum approaches. Similar difficulties have been experienced when using continuum approaches to model turbulent fluid flows, foams, rock falls and explosive events [8–10]. For such problems, the so-called discrete methods have been developed and are often preferred. While continuum approaches are useful and valuable, they may not be the best or optimal way to integrate biological information.

Discrete approaches have been gaining traction for modelling the behaviour of collections of cells [11–13]. In these approaches each cell is represented by a discrete ‘agent’, often a circular (2D) or spherical (3D) object that can move in response to external forces. These approaches have been used to model a range of tissues and their behaviour, such as colon crypt homeostasis [14–16], epidermis [17], cancer [18–22], morphogenesis and patterning in development [23,24], as well as branching structures in angiogenesis. These techniques have been given many names, including agent-based modelling [25], discrete element methods (DEM, granular mechanics) [26], molecular dynamics simulations [27] and smoothed particle hydrodynamics (chemistry, chemical engineering, fluid dynamics) [28], but when simulating mechanical behaviour they are all based on the idea of solving Newtonian physics on an ensemble of discrete particles. The advantage we see in this approach is that complex behaviour can emerge from relatively simple rules applied at the agent level. That is, there is arguably less abstraction than in the partial differential equation—finite element method (PDE-FEM) approach.

In this paper we present a discrete-element agent-based modelling environment to model cell-cell and cell-ECM mechanical interactions. In contrast to the overwhelming majority of agent based models of biological tissues which use a single discrete agent to represent each cell, we propose to represent cells and ECM using multiple agents. This has the immediate advantage of increasing the simulation resolution, allowing large deformation of individual cells (e.g. to model cell spreading or epithelial-to-mesenchymal transitions), realistic cell-cell and cell-ECM physical interactions and a more nuanced control of cell behaviour, which is difficult in a continuum mechanics approach. The main disadvantage is an increase in computational expense; nevertheless, the proposed solution method is completely explicit and therefore perfectly suitable for parallel implementation on Graphics Processing Units, using techniques similar to those presented in [29]. We will demonstrate the flexibility of the proposed agent-based model on a number of biological problems of intense ongoing interest and compare it to competing simulation methods in order to demonstrate its benefits.

## Methods

### Proposed discrete framework for modelling biological systems

In the proposed discrete-particle, agent-based framework a whole cell is represented by multiple 'agents' or ‘particles’, each of which represent a portion of the cell's matter. This allows the freedom for each agent to have its own set of parameters, qualities, and rules for interacting with other agents. For example, at its most basic level, one group of agents could represent the cytoplasm, while another group of agents represents the cell's membrane. For a given set of distinct rules given to these agents, the interactions and emergent behaviour of the collection of agents can be observed. In this way, selecting the best model for a cell can be accomplished by comparing different agent rules and the emergent behaviour that follow.

One of the most important aspects of this approach is that each particle (agent) keeps track of the mechanical forces acting upon it, and thus the agents can be displaced within the simulation space as a result of these forces. This movement can be visualized in animations, which represent the deformation of the cell as a result of internal and external forces acting upon discrete particles that make up the cell.

**Fig 1:**
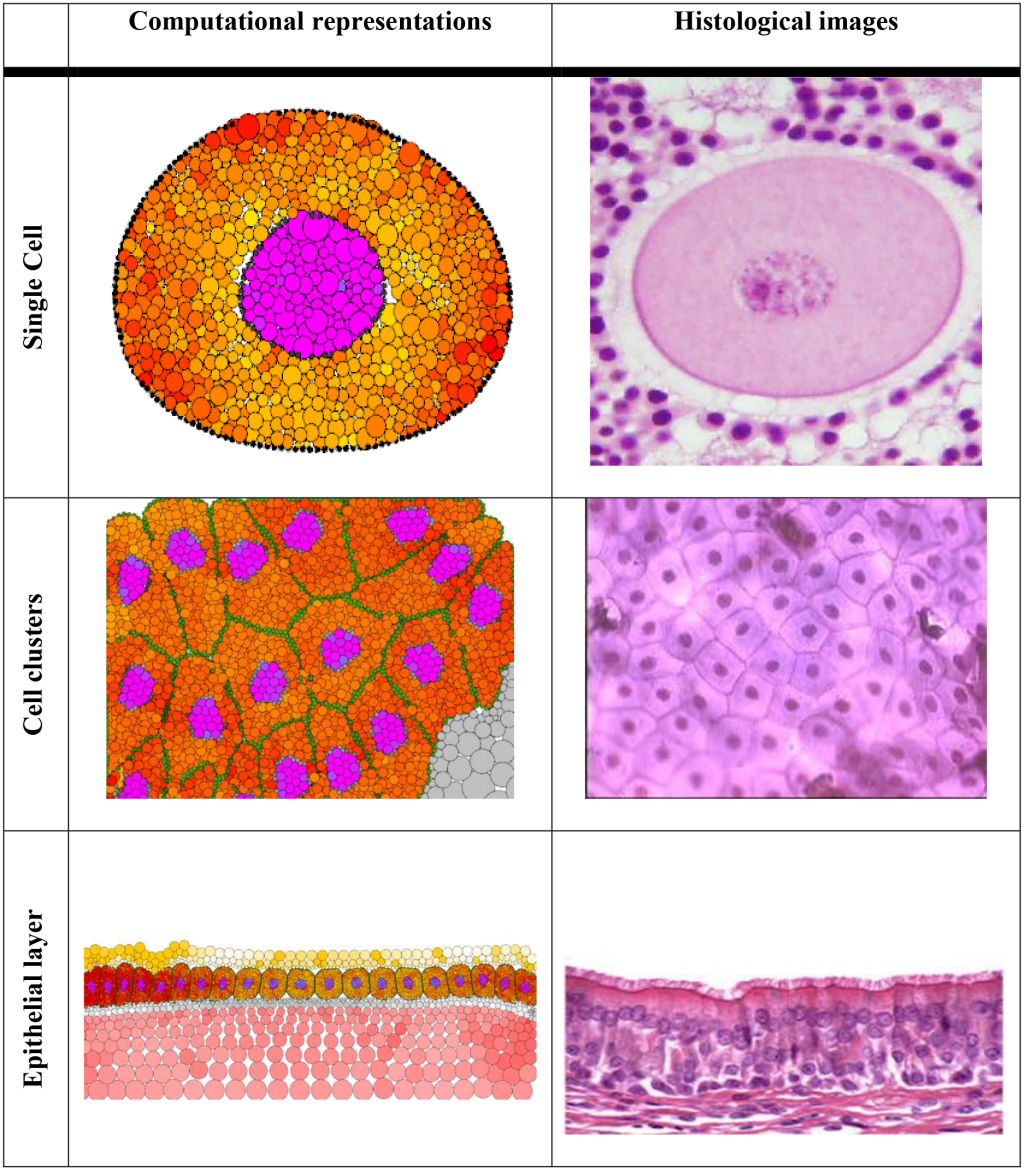
Computational representations and analogous histological images. Simulations can be developed at various scales, from a single cell to cell clusters and complex tissues. Cells and tissues are modelled using multiple particles (agents) which interact to each other; the macroscopic tissue behaviour emerges from these inter-particles interactions.

Some examples of biological tissue representation in the proposed framework are presented in Fig 1. A single cell with a nucleus can be constructed using cytoplasm, membrane, nucleus and nuclear membrane particles. Many such cells can self-organise, due to strong cell-cell adhesion between membrane particles and internally and externally applied forces, to form a cell cluster reminiscent of a typical tissue sample. At a higher level of detail, cells can interact with various types of extracellular matrix particles representing a basement membrane, mucus or stroma, to simulate, for example, the epithelial lining of the intestine.

Different properties of a cell can be created via suitable adjustment to the various properties of the component agents. External properties, such as basement adhesion and stromal stiffness, can be similarly recreated. Finer scale structures and processes can be represented by having more and smaller agent-based particles in a simulation. While there have been extraordinary advances in digital computers, which now permit the representation of tissues by many millions of particles, it remains important to do the computations as efficiently as possible. For this reason, multiscale agent-based discrete models may be developed. In this approach, a region of tissue may be represented generally using large particles, but particular regions of the tissue where more information is required may be represented using smaller particles, which provide higher local resolution. As importantly, for such particle-based methods, the position, material and biochemical properties are only stored at the particles rather than at every node in a spatial grid, avoiding unnecessary computations required for re-generating the grid every time the geometry of the cell structure changes. This allows the optimization of storage and reduction of processing bandwidth, enabling efficient parallelization of computations.

### Particle interactions for a cell

**Fig 2:**
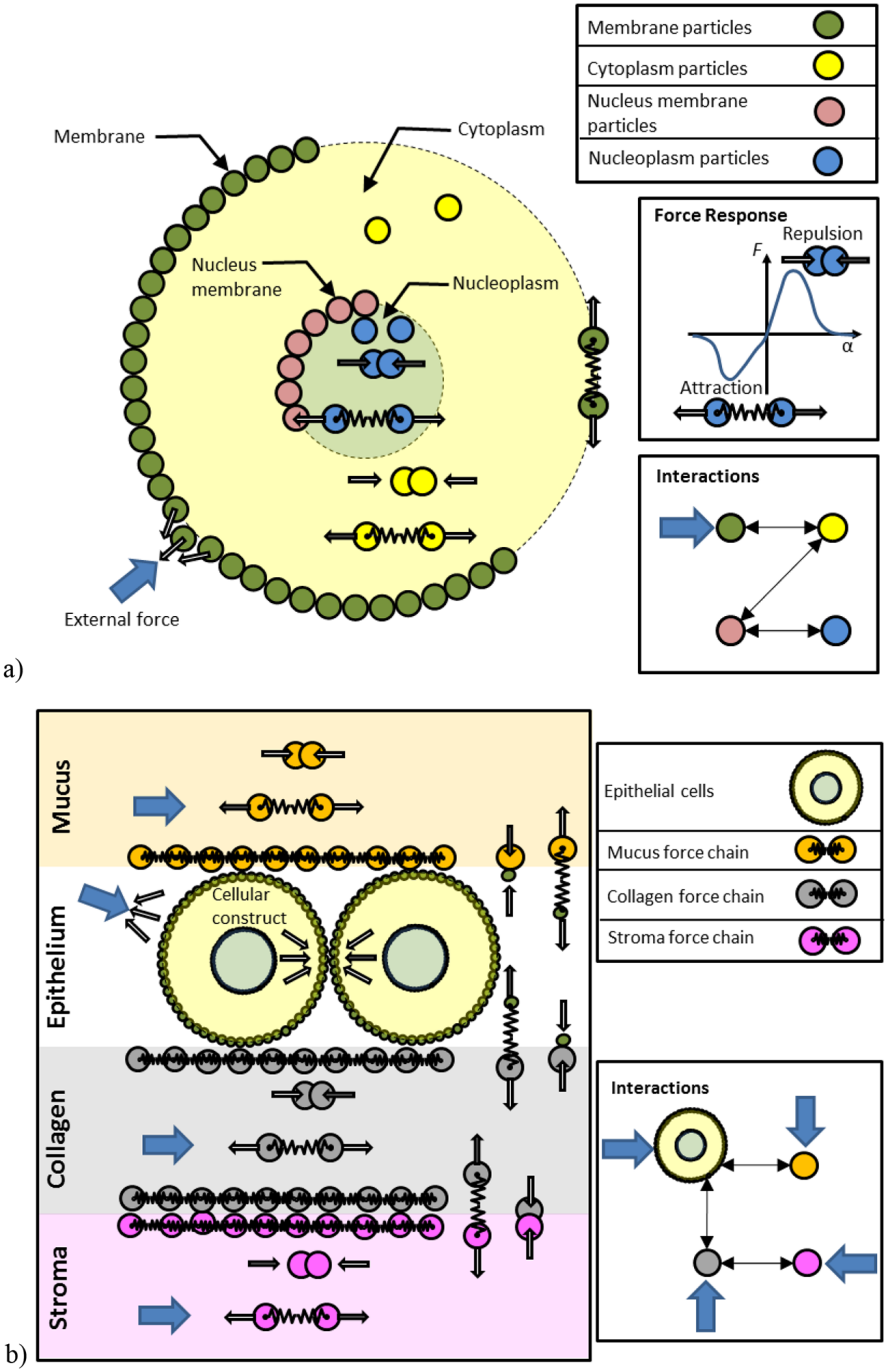
Representations of cells and extracellular components in the proposed discrete element framework. a) Example cell structure and possible interactions. b) Example tissue structure and possible interactions. The behaviour of each particle is influenced by its neighbouring particles through short range interaction forces.

The cell models described here include 4 cell particle types: cell membrane (with cell cortex) (M), cytoplasm (C), nuclear membrane (NM), and nucleoplasm (N), as shown in Fig 2a. We also consider the potential for particle interactions with a mechanical testing plate (P) and a basement membrane (BM). Therefore the number of potential cell particle interactions is 18. However, we have chosen our model parameters (via a series of initial parametric studies) to limit inter-compartment transport of nucleoplasm or cytoplasm particles. Therefore, as shown in Table 1, there are only 7 unique particle-particle interactions within a cell and an additional 2 external cell interactions (i.e. to plate or basement membrane). These interactions define our fundamental cell unit. More complicated tissue structures can be built by combining together multiple cells and ECM, as shown in Fig 2b.

**Table 1:** Interaction matrix for the constituents of a cell.

	M	C	NM	N	P	BM
**M**	X	X	-	-	X	X
**C**	X	X	X	-	-	-
**NM**	-	X	X	X	-	-
**N**	-	-	X	X	-	-

We consider only short-range normal interaction forces between particles, which we model as a nonlinear spring [26]. The elastic (spring) interaction force acting on particle *I* due to particle *J* is defined as:

$$F_{I-J}=\left[F_{I}^{a}-P_{I-J}\left(\alpha_{I-J}\right)\right]n_{I-J}$$ (1)

where $F_{I}^{a}$ is a constant attractive force between particles of the same type (M, C, NM, N, P, or BM) and *P*_*I-J*_ is a force dependent on the overlap/separation between particles, defined as the sum of particles’ radii minus the distance between the particles’ positions:

$$\alpha_{I-J}=\left(R_{I}+R_{J}\right)-||x_{I}-x_{J}||$$ (2)

and **n**_*I-J*_ is the unit vector from the position of particles *I* towards the position of particle *J*:

$$n_{I-J}=\frac{x_{J}-x_{I}}{\left||x_{I}-x_{J}|\right|}$$ (3)

We chose the force *P*_*I-J*_ as a simple linear attractive force when particles are separated (up to a maximum separation distance) [26] and as a Hertzian contact between two elastic spheres [30] when the particles penetrate each-other:

$$P_{I-J}\left(\alpha_{I-J}\right)=\{\begin{array}{cc} 0, & -\delta_{I}>\alpha_{I-J}\\ k_{I-J}^{a}R_{I-J}\alpha_{I-J}\;, & 0>\alpha_{I-J}\geq -\delta_{I}\\ k_{I-J}^{r}\sqrt{R_{I-J}}\alpha_{I-J}^{3/2}\;, & \alpha_{I-J}\geq 0 \end{array}$$ (4)

The elastic force and the attractive force are zero if particle *J* is outside the influence radius of particle *I*, −*δ*_*I*_ > *α*_*I-J*_ (have limited range).

The spring constants and equivalent radius appearing in eq (4) are computed based on the individual properties of the two particles:

$$R_{I-J}=\frac{R_{I}R_{J}}{R_{I}+R_{J}}$$ (5)

$$k_{I-J}^{a}=\frac{k_{I}^{a}k_{J}^{a}}{k_{I}^{a}+k_{J}^{a}}$$ (6)

$$k_{I-J}^{r}=\frac{4}{3}{\left(\frac{1-\upsilon_{I}^{2}}{E_{I}}+\frac{1-\upsilon_{J}^{2}}{E_{J}}\right)}^{-1}$$ (7)

where *E*_*I*_ and *υ*_*I*_ represent the Young’s modulus and Poisson’s ratio associated with particle *I* [30] and $k_{I}^{a}$ is the linear spring constant for attraction associated with particle *I*. The forces generated during interactions with linear objects (such as the plate or basement membrane) are computed by considering such objects as spheres with infinite radius.

A viscous drag force acting on a particle *I* due to particle *J* is defined based on the difference in particles’ velocities (only for the *n*_*I*_ particles within a given distance to particle *I*), as:

$$D_{I-J}=\{\begin{array}{ll} \;0, & -\delta_{I}>\alpha_{I-J}\\ -\beta_{I}/n_{I}\left(v_{I}-v_{J}\right), & \;\alpha_{I-J}\geq -\delta_{I} \end{array}$$ (8)

### Equation of motion

The total force acting on particle *I* due to its neighbouring particles is obtained by summing the influences of all surrounding particles:

$$F_{I}=\underset{J\neq I}{\sum^{\;}}\left(F_{I-J}+D_{I-J}\right)$$ (9)

Because only short range interactions are consider, the summation in eq (9) has non-zero terms only for *n*_*I*_ neighbouring particles, as resulting from eqs (4) and (8). We will name *N*_*I*_ this set of neighbouring particles.

The motion of particle *I* is governed by Newtonian physics [26]:

$$m_{I}{\ddot{x}}_{I}=\underset{J\in N_{I}}{\sum^{\;}}\left(F_{I-J}+D_{I-J}\right)+F_{I}^{E}$$ (10)

with the externally applied force $F_{I}^{E}$ including any other forces other than particle interactions (such as gravity).

The total drag force acting on a particle *I* due to all its neighbouring particles becomes, taking into account eq (8), as

$$D_{I}=\underset{J\in N_{I}}{\sum^{\;}}D_{I-J}=-\beta_{I}\left(v_{I}-\underset{J\in N_{I}}{\sum^{\;}}v_{J}/n_{I}\right)$$ (11)

and is therefore proportional to the difference between the velocity of particle *I* and the average velocity of the surrounding particles which exercise an influence on particle *I*. The chosen form for the drag force, which originates from multiple dissipative processes taking part at cellular level, is different from the form used in other discrete element simulations, where it only acts in the normal direction between particles [26]. This drag force resembles more a viscous drag, as it is proportional to the velocity of the reference particle relative to its surrounding particles. More complex constitutive equations can be employed to represent particular dissipative processes as required.

### Numerical solution

The equation of motion for a given particle *I* can be rewritten, considering the definition of forces involved, as:

$$m_{I}{\ddot{x}}_{I}+\beta_{I}\left({\dot{x}}_{I}-\underset{J\in N_{I}}{\sum^{\;}}{\dot{x}}_{J}/n_{I}\right)+\underset{J\in N_{I}}{\sum^{\;}}P_{I-J}\left(x_{I},x_{J}\right)n_{I-J}=F_{I}^{E}+\underset{J\in N_{I}}{\sum^{\;}}F_{I-J}^{a}n_{I-J}$$ (12)

The equations of motion for all particles in the model lead to a system of coupled second degree differential equations. This system of equations can be solved using a numerical integration method. We chose to use the Verlet integrator [31] for numerical integration. One deficiency of the Verlet integrator is the fact that it cannot handle velocities in the equation of motion. To overcome this limitation, we use in the time stepping procedure velocities estimated one time step behind the displacements. This also allows the decoupling of the equations of motion and results in an explicit integration algorithm, better suited for parallel implementation. While accurate inclusion of velocities would be needed in dynamic simulations, most biological simulations are quasi-static, and therefore viscous damping is only used to increase the stable time step and stabilise the numerical solution.

An explicit integration procedure requires the time step used to be less than a critical time step. The critical time step is computed based on the properties of each pair of particles forming a basic spring, considering the mass of the particles, the stiffness of the system (the maximum stiffness in case of a non-linear system) and the damping as shown in [26]. The used time step is the computed as the critical time step multiplied with a subunitary safety factor. In dynamic simulations this safety factor must be chosen very low to guarantee solution accuracy. For quasi-static simulations, as those presented in this paper, a much higher safety factor can be used, close to one, as the time step is only chosen based on stability considerations [32].

We implemented the presented computational framework in java using the Repast Simphony agent-based simulation tool-kit [33].

## Results

This section contains several computational results obtained using the proposed framework. These results demonstrate the flexibility of the framework, with the macro-scale cell behaviour being controlled using a few easy to understand model parameters. Unless specified otherwise, the basic model parameters used in the simulations are presented in Table 2. These parameters were calibrated to reproduce the experimental stress-strain behaviour of an isolated nucleus and of a whole cell in an unconfined compression plate-load test [3].

**Table 2:** Basic model parameters.

Parameter	Symbol	Unit	Particle			
			M	C	NM	N
Average radius	*R*_*I*_	(×10^−6^) m	0.15	0.25	0.15	0.25
Attractive force	$F_{I}^{a}$	N	1×10^−10^	5×10^−11^	1×10^−10^	5×10^−11^
Influence radius	*δ*_*I*_	(×10^−6^) m	2	2	2	2
Spring constant	$k_{I}^{a}$	N/m	1×10^−8^	1×10^−8^	1×10^−8^	1×10^−8^
Young’s modulus	*E*_*I*_	Pa	1×10^7^	1×10^3^	1×10^7^	5×10^3^
Poisson’s ratio	*υ*_*I*_	-	0.5	0.5	0.5	0.5
Drag factor	*β*_*I*_	kg/s	0.05	0.05	0.05	0.05

### Cell initialisation

Before creating higher order cell structures, we need to make sure that we can create single cells with the desired properties. We have therefore initialised single cells and studied the influence of model parameters on their behaviour.

**Fig 3:**
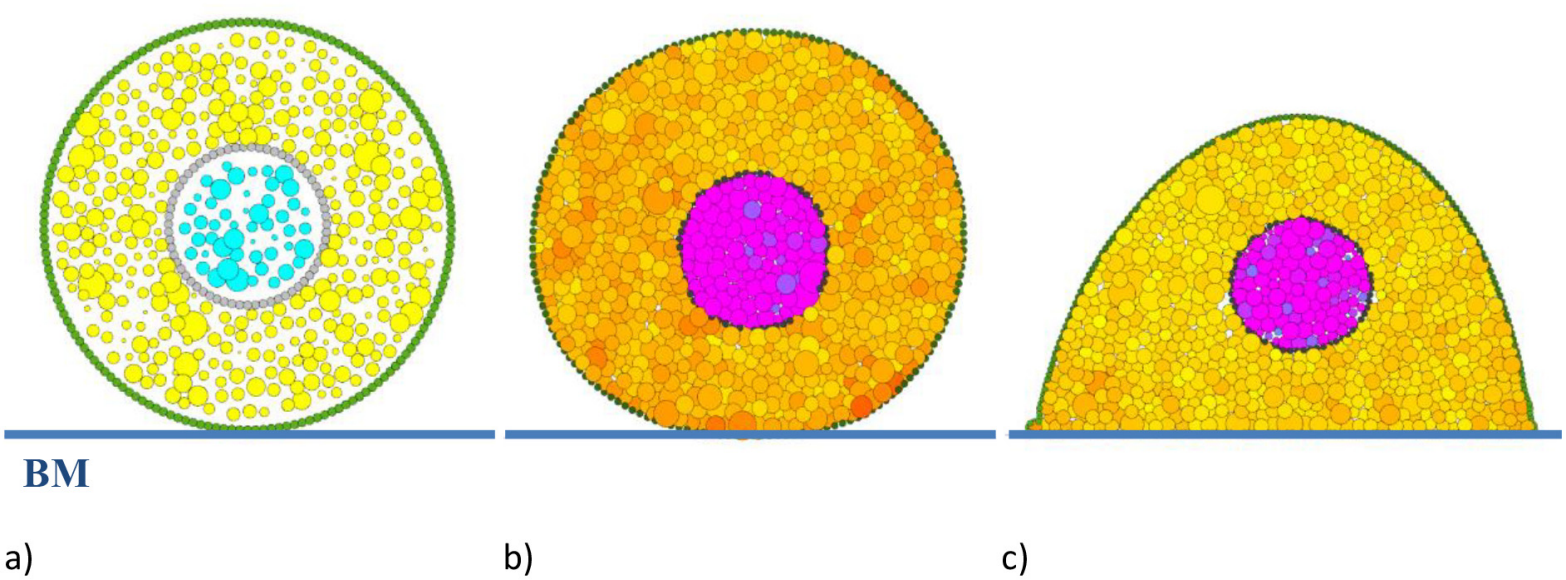
Cell initialisation procedure. a) Initial placement of particles defining the different parts of the cell. b) Cell after the initialisation procedure when no adhesion to the boundary is used. c) A spread cell geometry can be created by using attraction of membrane particles to the basement membrane (BM). The spread cell geometry depicted was achieved using a spring constant $k_{BM}^{a}$ = 1×10^−11^ N/m and an influence radius *δ*_*BM*_ = 3μm for the BM. We have used a colour scale to indicate the net contact force experienced by each of the cell’s internal particles—the darker the colour the higher the force.

The first step in initialising a cell is the creation of the particle distribution. Different algorithms can be used to create an initial particle assembly [30]. The initial cell shape varies depending on problem. In all the presented simulations we start with round cells. We initiate the creation of the cell by defining the position of the cell and nuclear membranes and populate them with equally spaced particles. We then populate the cytoplasm and nucleoplasm with the corresponding particles of initial radius *R*, at random positions, but with a number significantly less than the final target particle number *N* in each compartment (specifically we begin with *N/$\sqrt{2}$* particles). The cytoplasm and nucleoplasm particles are then allowed to slowly grow (with a linear growth rate over time) and divide (by adding another particle at the same location) upon reaching $\sqrt{2}\;$ *R* until a predefined threshold is reached in the cell membrane tension caused by the separating neighbouring membrane particles. To avoid crystalline packing that can occur with particles of the same size, a variance of 30% in the original particle radius was introduced. A fully developed cell after this process is illustrated in Fig 3.

A round cell can be readily achieved by the method described above (Fig 3b). However a spread cell (Fig 3c) requires additional steps. Specifically, a basement membrane (BM) is needed and an attraction force defined between the cell and the BM. As the cell becomes less round there is an increase in the membrane length and tension [34]. In the initialisation step we allow this tension to relax by adding additional membrane particles. Specifically a new membrane particle is added between any two neighbouring membrane particles separated by a gap of greater than half of a membrane particle size. This approach is consistent with experimental data showing that a cell responds to alterations in membrane tension by adjusting its overall membrane area [34].

**Fig 4:**
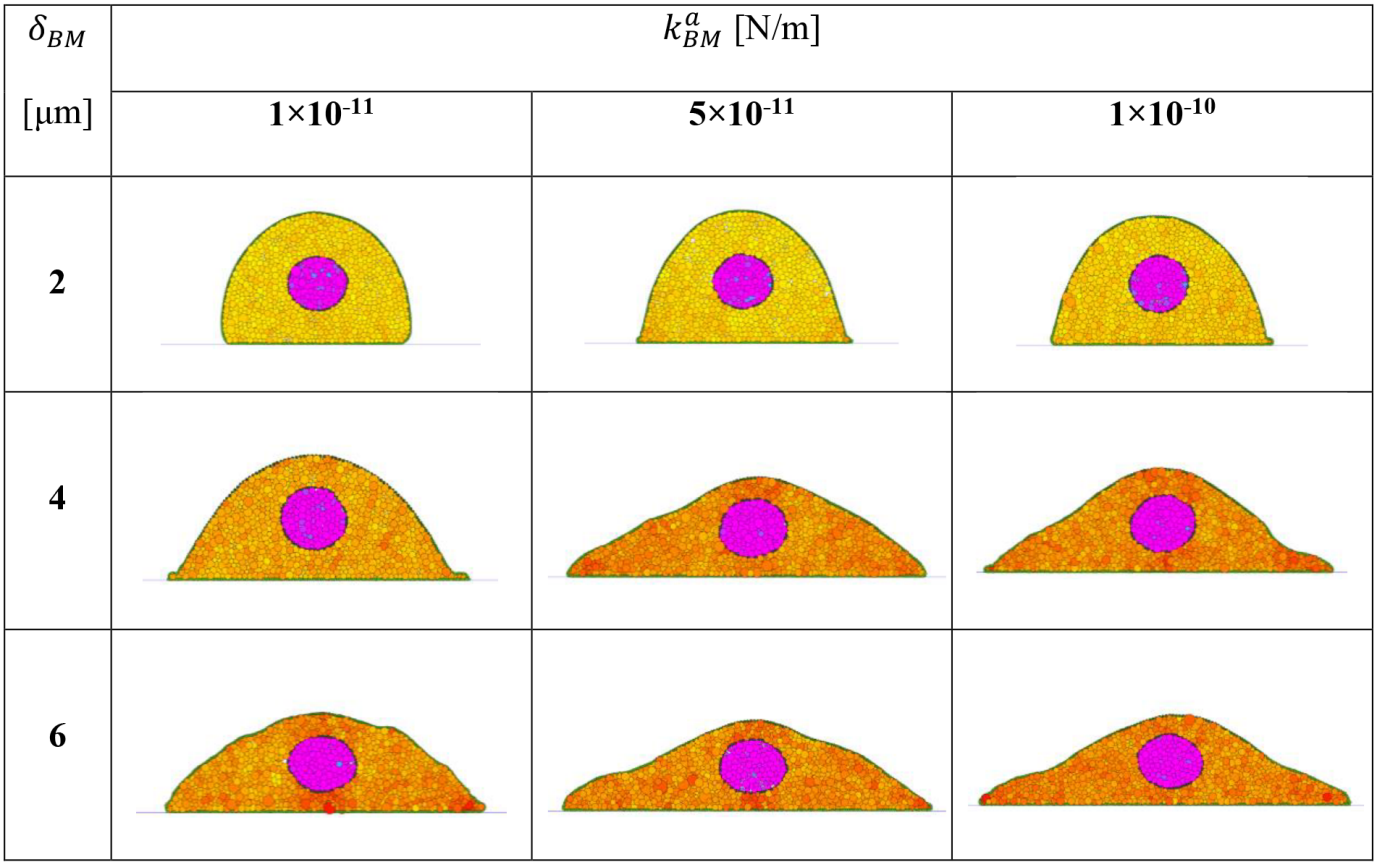
Influence of BM parameters on cell spreading. The attraction of membrane particles to the BM is characterised by both a linear attractive force (adhesion) spring constant and an influence radius. These quantities correspond to, for example, the extension distance of filopodia to a surface and the force generated by the filopodia. The influence radius *δ*_*BM*_ has a greater influence on the geometry of the cell as compared to the spring constant $k_{BM}^{a}$.

In the simulation the attraction of membrane particles to the BM is characterised by both a linear attractive force (adhesion) spring constant and an influence radius. These quantities correspond to, for example, the extension distance of filopodia to a surface and the force generated by the filopodia, although many aspects affecting cell attachment (cell behavior, material surface properties, and environmental factors) is captured by these parameters [35]. Changing the adhesion force spring constant and influence radius modifies the degree of spreading of a cell. The effect of changes in these parameters is shown in Fig 4.

The morphological properties of cells are fundamental to quantitative cytology. Our results demonstrate that cell spreading on a BM can be simulated by adjusting properties associated with the BM.

### Mechanical response of a cell

The mechanics of single cells has been investigated experimentally using a number of standard tests, including parallel plate compression [3] and micropipette aspiration [36–38]. These various methods, on a range of cells, have found that the cell nucleus is generally 1–20 times stiffer than the cytoplasm. Typically the force-displacement relationship of each is non-linear, the stiffness increasing with strain. Interestingly, the apparent stiffness of the cell depends on the ‘spreading’ of the cell, stiffness increasing with cell spreading [3].

We will demonstrate how our cell model can be calibrated to reproduce experimental data from microplate compression tests. The calibration will be performed in a bottom-up fashion, with the nucleus parameters being determined first, followed by the cytoplasm and membrane parameters. This way only a limited number of parameters are determined at each step. Caille et al. [3] performed strain-controlled compression of single cells between parallel plates with the top plate moving at a velocity of 0.25μm/s. He recorded force-displacement relationships for isolated nuclei, single whole rounded cells and whole spread cells. Although the data for the mechanical and physical properties is for endothelial cells, the values are typical of a wide range of cells including epithelial cells, as shown by several reviews [3,36–38].

Experimental cell diameters were in the range of 10 to 15 μm, with a nucleus diameter range of 3 to 7 μm. In our model we created a cell having a 15 μm diameter with a 6 μm nucleus. The computational domain for a single cell is represented by 981 circular discrete elements (726 cytoplasm, 155 membrane and 100 nucleus discrete elements).

The parameters of the cell model can be divided into three categories: parameters that define the elastic properties (Young’s modulus, attractive force and spring constant for nucleus and cytoplasm), parameters defining the dynamic behaviour (viscosity) and parameters that control the integrity of the cell (the spring constant of the membrane). Because the simulated experiments are quasi-static, the viscosity was mainly used to increase the critical time step and simulation stability. The spring constant of the membrane was chosen high enough to prevent seepage at the maximum deformation.

We start the calibration of our cell model by first identifying the parameters of an isolated nucleus based on the available experimental data. The calibrated nucleus is then placed within either a round or a spread cell. The nucleus is categorized as a much stiffer organelle in comparison with the cellular cytoplasm, and acts as an important compression-bearing material within the cell [39].

We simulated the strain-controlled compression experiments, with the total force acting on the moving plate used for direct comparison to the experimental data. Because our simulations are in 2D, while the experimental tests are in 3D, we extended our results to 3D by considering, in a first approximation, that the axisymmetric 3D object is equivalent with a collection of independent, identical 2D sections rotated around the symmetry axis. Therefore, the total force acting on the compression plate was computed as the sum of the contributions *F*_*I*_ from all the membrane particles *I* contacting the plate weighted with the number of particles that would be found at the position of particle *I* relative to the position of the axis of symmetry on the plate **x**_*C*_ when particle *I* is rotated to form a circle of particles around the axis of symmetry:

$$F_{plate}=\underset{I}{\sum^{\;}}F_{I}\frac{2\pi ||x_{I}-x_{C}||}{R_{I}}$$ (13)

**Fig 5:**
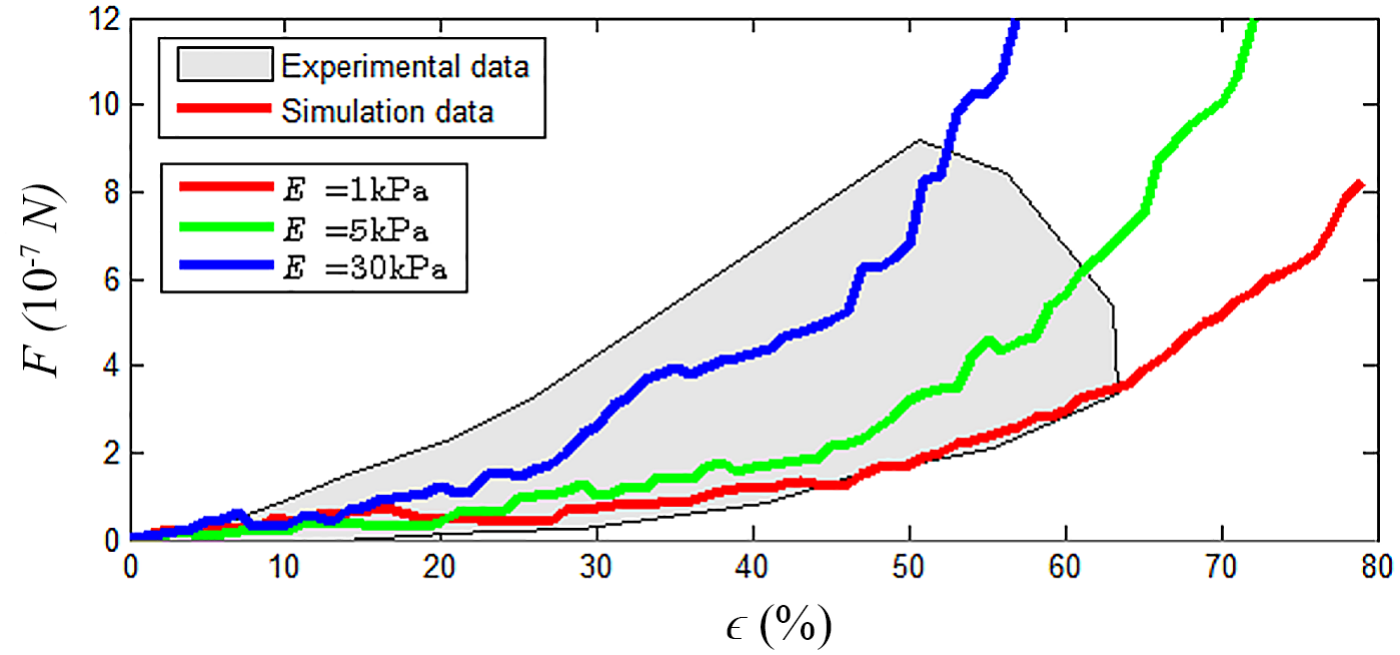
Cell nucleus calibration. Comparison between experimental and simulation results for different values for Young’s modulus of the nucleus particles. Experimental data taken from Caille et al. [3]. The behaviour of the nucleus is well represented by the chosen force expressions and corresponding fitted parameters over a large range of deformation.

The nucleus calibration results are presented in Table 3 and Fig 5. While the main parameter influencing the stiffness of the nucleus is the Young’s modulus of the constitutive particles, when Young’s modulus is modified other parameters need to be scaled accordingly (for example to prevent particle seepage through the cell membrane).

**Table 3:** Nucleus calibration parameters used in the simulations.

Parameter	Symbol	Unit	Young’s modulus *E*_*N*_, *E*_*NM*_ [kPa]		
			1	5	30
Attractive force	$F_{N}^{a}$	N	5×10^−12^	5×10^−12^	2.5×10^−11^
Spring constant	$k_{NM}^{a}$	N/m	1×10^−8^	1×10^−8^	5×10^−8^
Drag factor	*β*_*N*_	kg/s	0.003	0.003	0.01

**Fig 6:**
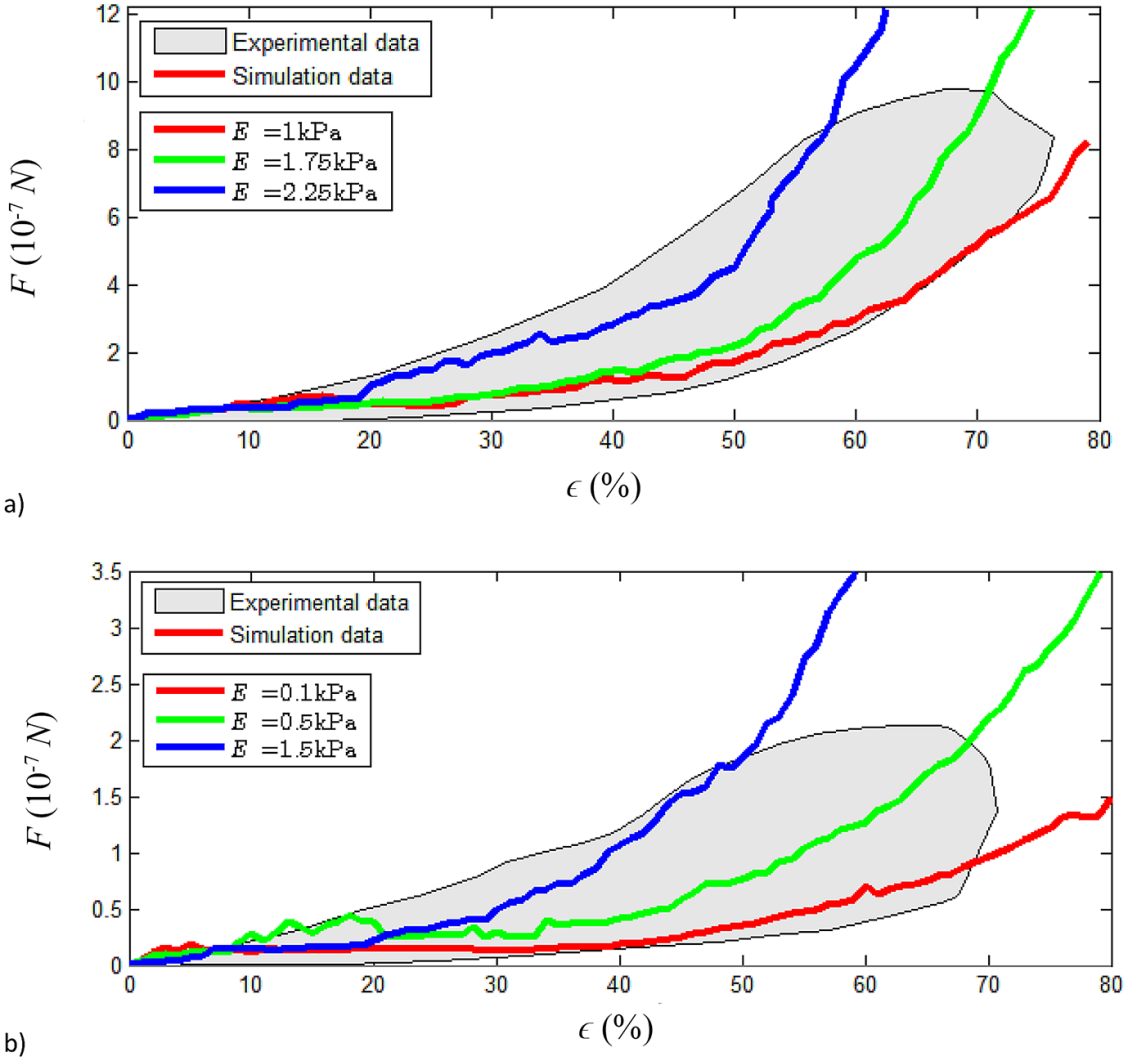
Cell calibration. Comparison between experimental and simulation results for different values for Young’s modulus of the cytoplasm particles. a) Spread cell. b) Round cell. Experimental data taken from Caille et al. [3]. The behaviour of the cells is well represented by the chosen force expressions and corresponding fitted parameters over a large range of deformation.

The cell calibration results are presented in Table 4 and Fig 6 for spread and round cells. The stiffness of the nucleus was matched with the desired stiffness of the cell (stiffer nucleus used in a stiffer cell, as specified in Table 4).

**Table 4:** Upper and lower limits of cell calibration parameters used in the simulations.

Parameter	Symbol	Unit	Young’s modulus *E*_*C*_, *E*_*M*_ [kPa]			
			Spread cell		Round cell	
			1	2.25	0.1	1.5
Attractive force	$F_{C}^{a}$	N	2.5×10^−12^	5×10^−12^	5×10^−13^	2.5×10^−12^
Attractive force	$F_{M}^{a}$	N	5×10^−11^	1×10^−10^	1×10^−11^	5×10^−11^
Spring constant	$k_{M}^{a},\;k_{NM}^{a}$	N/m	5×10^−9^	1×10^−8^	1×10^−9^	5×10^−9^
Young’s modulus	*E*_*N*_	kPa	1	25	1	25
Drag factor	*β*_*I*_	kg/s	0.002	0.003	0.001	0.002

As indicated by the grey-shaded region shown the above figures, the experimental force-displacement data shows considerable variation. Nevertheless, model parameters can be found to reproduce the observed behaviour of the nucleus and the whole cell, in both the round and spread configurations. Although the Young’s moduli *E*_*N*_ and *E*_*C*_ correspond to particles’ stiffness rather than to ‘continuum’ stiffness observed in experiments, their ratio is similar to the ratio of nucleus to whole cell stiffness observed in experiments. The apparent stiffness of the cell in the compression experiments varies between round and spread cells [3]. Our simulations reproduce this behaviour, showing stiffer spread cells for similar particle material properties.

**Fig 7:**
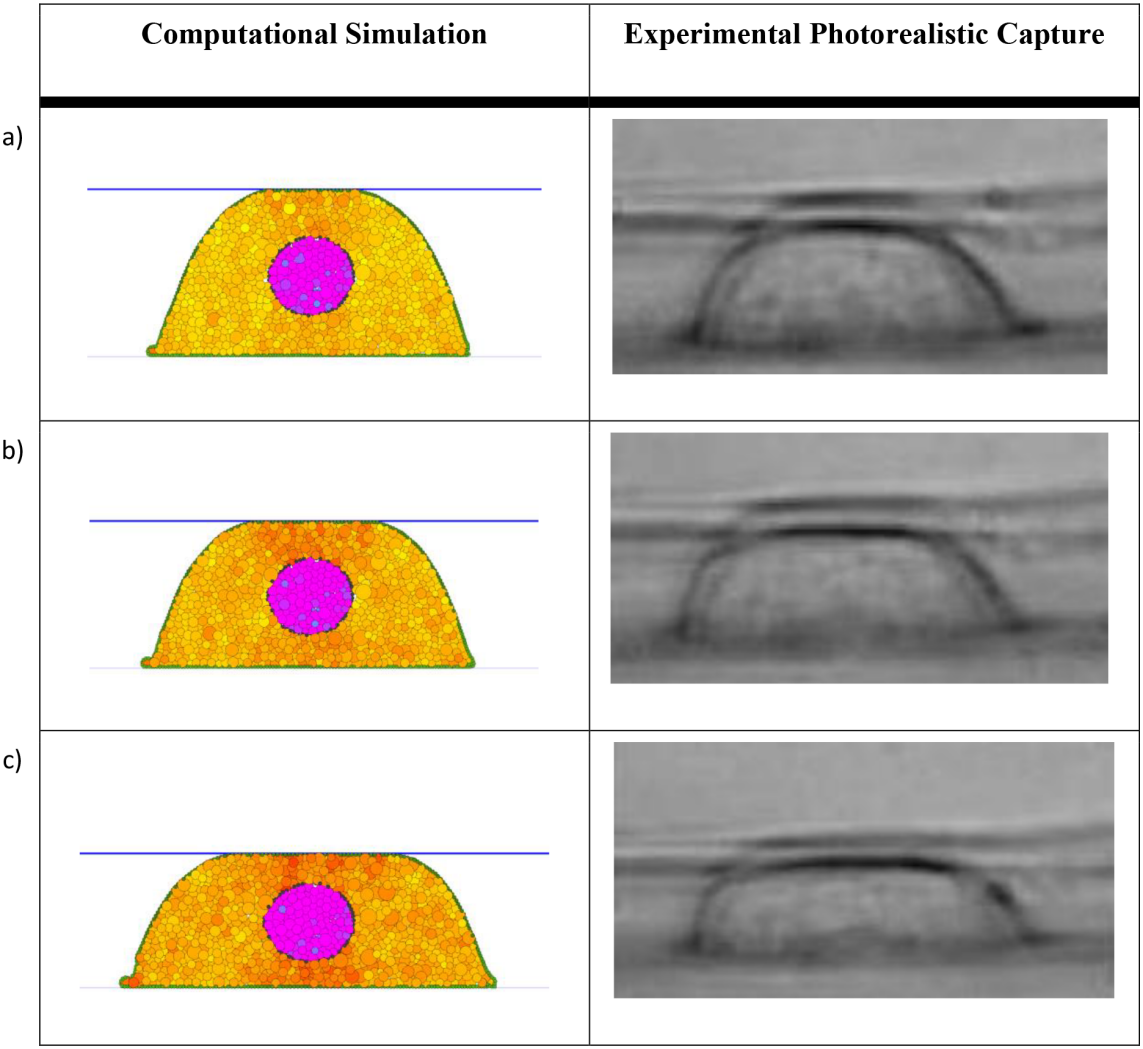
Spread cell compression based on *E*_*C*_ = 2.5 kPa and *E*_*N*_ = 25 kPa. The computational simulation results for a) 10%, b) 20% and c) 30% compression are compared against the experimental results from [3]. The spread cell geometry depicted was achieved using a spring constant $k_{BM}^{a}$ = 1×10^−11^ N/m and an influence radius *δ*_*BM*_ = 3μm for the BM. Very good match between the simulation and the experimental results can be observed.

**Fig 8:**
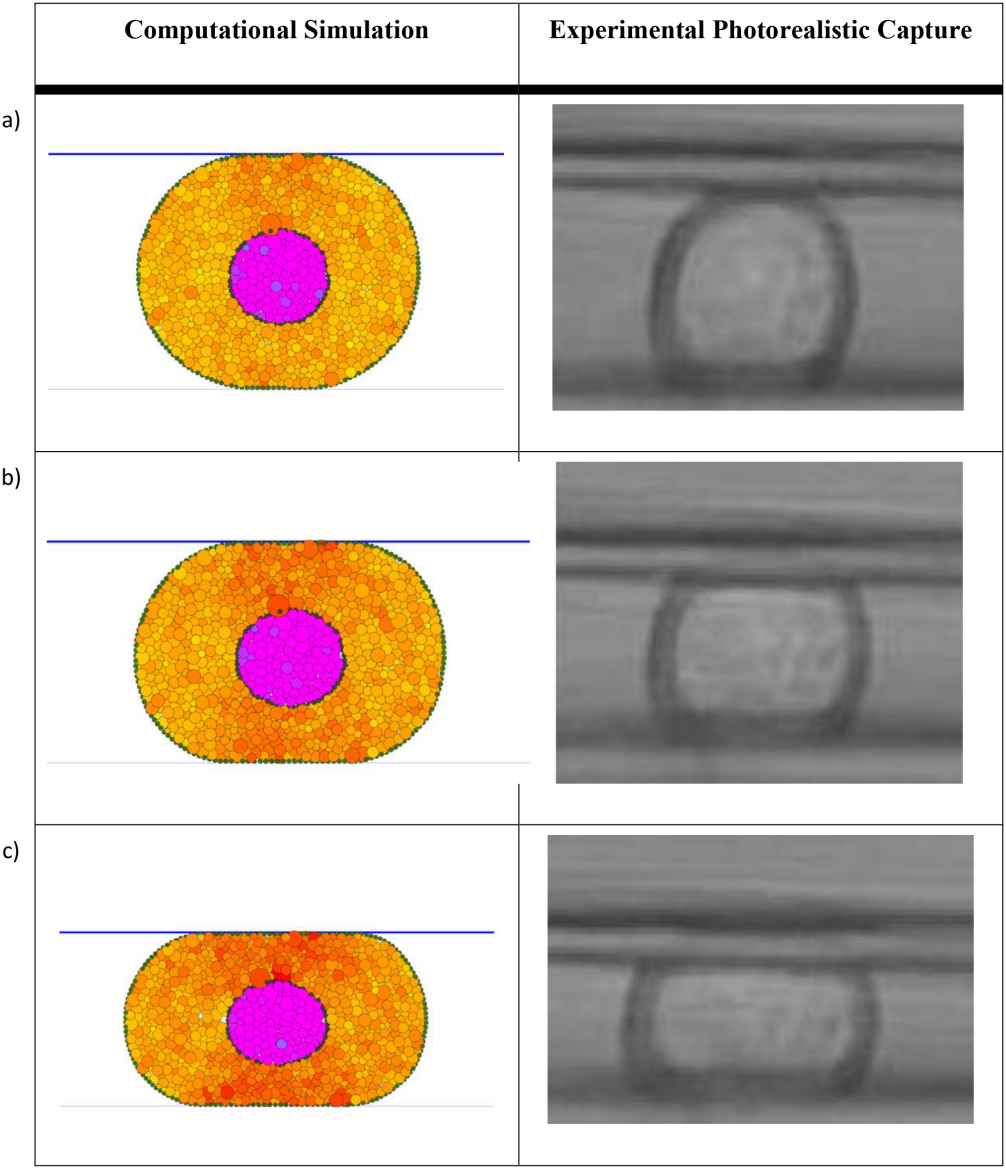
Round cell compression based on *E*_*C*_ = 1.0 kPa and *E*_*N*_ = 25 kPa. The computational simulation results for a) 10%, b) 20% and c) 30% compression are compared against the experimental results from [3]. Very good match between the simulation and the experimental results can be observed.

A comparison between the simulated compression experiments and the experimental results are presented in Fig 7 for a spread cell and in Fig 8 for a round cell. We notice the very good qualitative match between the simulation and the experimental results in terms of cell deformation behaviour.

### Comparison to previous modelling approaches

Key applications of modelling and simulation in computational biology are the development of explanatory models for both generating and testing of new hypothesis. In many such cases there are no quantitative experimental data that can be used for comparison to the predictions of the model; nevertheless, there are competing modelling approaches which provide some data for validating a new model. In this section we demonstrate how the proposed modelling framework can be used to solve several cell biomechanics problems and compare our results to those obtained using competing modelling approaches.

### Regulating cell shape in epithelial layers

**Fig 9:**
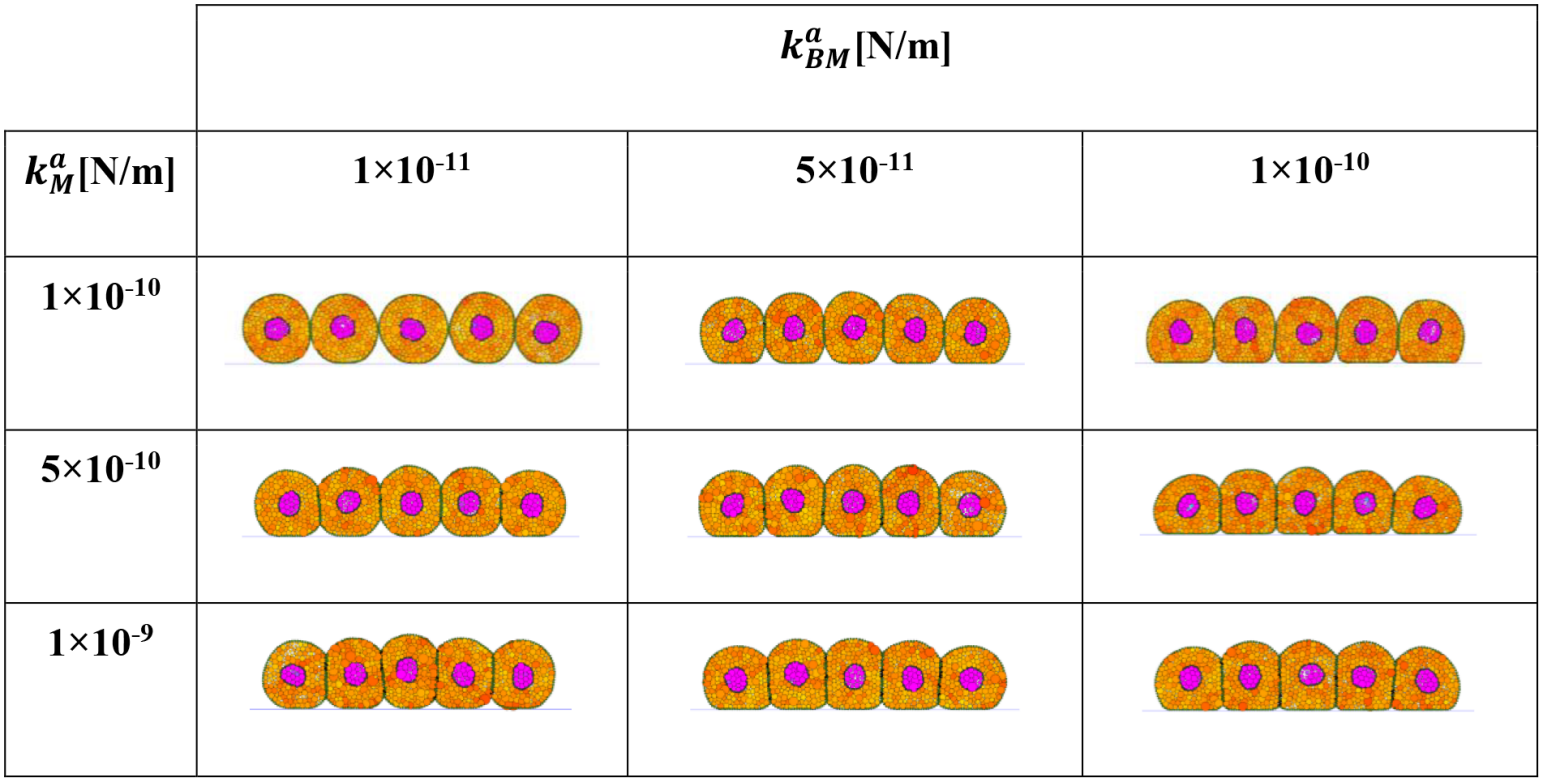
Influence of membrane and basement membrane spring constants on cell shapes in an epithelial layer. In all cases the influence radii are *δ*_*BM*_ = 2μm and *δ*_*M*_ = 0.2μm. Increasing the adhesion to the basement membrane ($k_{BM}^{a}$) leads to spread cells, while increasing adhesion between cells ($k_{M}^{a}$) leads to cells with a columnar appearance.

These experiments demonstrate the possibility to regulate cell shape in a cell layer by changing the properties of membrane particles and basement membrane (Fig 9). Similar to the adhesion to the basement membrane, which regulates the spreading of cells, adhesion between cells (e.g. via cadherins) can be easily controlled by modifying the spring constant $k_{M}^{a}$ and an influence radius *δ*_*M*_ for the membrane. The influence radius *δ*_*M*_ for the membrane is set to be short (0.2 microns), reflecting the short-range interactions of membrane bound cadherin molecules. However, it could be increased to represent cell projections if required in the simulation.

Increasing the adhesion to the basement membrane leads to spread cells, while increasing adhesion between cells leads to cells with a columnar appearance. These results are in correlations with the results presented in [40], where cells in an epithelial sheet were modelled as hexagonal prisms. Nevertheless, while in [40] the shape of a given cell is obtained by minimizing a carefully constructed energy function, in our framework the shape of the cell is controlled by direct modification of the adhesion parameters and the interaction between cell’s membrane and the environment. Also, the modelling approach presented in [40] induces epithelial sheet deformation by modifying the shape of the cells, without considering the influence of external stresses. Our approach allows the computation of epithelial sheet deformation under external stresses, such as those induced by cell proliferation, as we will show in the following section.

### Epithelial sheet deformation

Proliferation and reorganisation of the cells in epithelial tissues has an important role in the biological functions of the human body including the process of early vertebrate development [41], precise regulatory process and renewal of the colonic epithelial cell wall [14], as well as pancreatic islet development [42]. The mechanical disturbance caused by epithelial cells pushing against one another is not well studied. This section presents simulation results of the behaviour of an epithelial layers of cells under compression (possibly by newly generated cells e.g. as might occur during migration of cells along a colonic crypt [14,43]). The compression leads to the reorganisation of cell particles, resulting in changes of the shape and position of the cells.

In many epithelial layers the connectivity between cells via cadherin and other mechanisms is critical to the function of the tissue, as it ensures the integrity of the epithelial barrier [44]. Cadherins are also known to be important for cell sorting and the epithelial-mesenchymal transitions. In the following simulation we investigate the effect of a loss or gain of cadherin cell-cell adhesion strength in an epithelial layer under mechanical stress, simply by adjusting the adhesion parameters. Later we will also explore the effect of the position of the nucleus within the cell on the deformation response of an epithelial layer.

**Fig 10:**
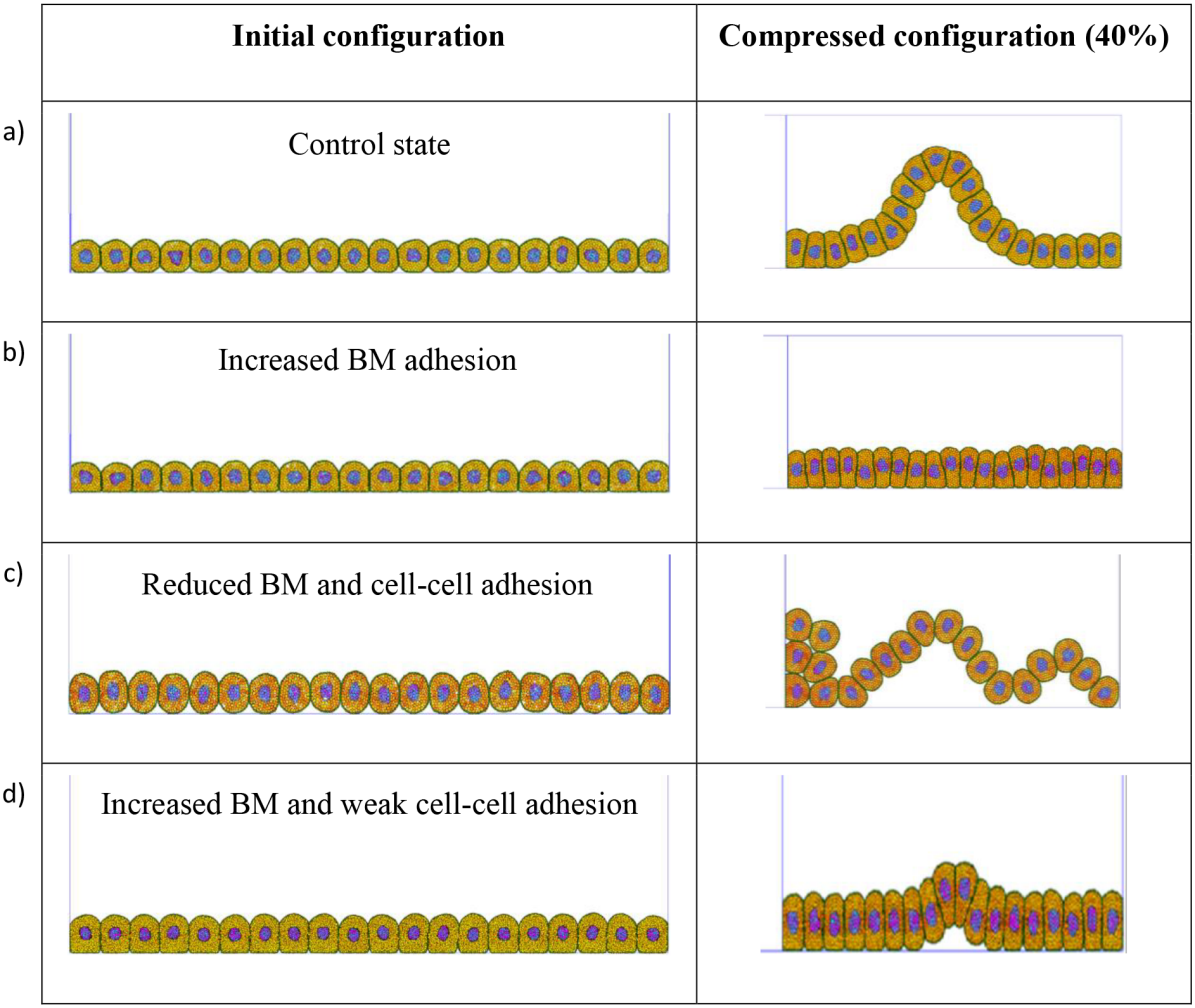
Deformation of a single epithelial layer. a) Control state ($k_{BM}^{a}$ = 1×10^−11^ N/m, *δ*_*BM*_ = 0.3 μm, $k_{M}^{a}$ = 5x10^-10^ N/m, *δ*_*M*_ = 0.2 μm). At 40% compression a single buckle emerges. b) By increasing the BM adhesion ($k_{BM}^{a}$ = 1×10^−10^ N/m, *δ*_*BM*_ = 0.4 μm) and using the same cell-cell adhesion parameters the buckling is suppressed. c) Reducing BM and cell-cell adhesion ($k_{BM}^{a}$ = 1×10^−12^ N/m, *δ*_*BM*_ = 0.2 μm, $k_{M}^{a}$ = 5x10^-12^ N/m, *δ*_*M*_ = 0.1 μm) causes the cell layer to cascade quickly and easily without any resistance. d) Increasing the BM adhesion and weakening the cell-cell adhesion ($k_{BM}^{a}$ = 5x10^-10^ N/m, *δ*_*BM*_ = 0.2 μm, $k_{M}^{a}$ = 5x10^-11^ N/m, *δ*_*M*_ = 0.1 μm) results in the budding of a cell from the layer of cells.

Our model consists of 20 cells placed on a basement membrane. The compression of this cell layer is simulated using a rigid vertical wall, initially on the far left, which moves from left to right, while a rigid vertical wall on the far right is kept static. The rate of compression used is 0.25 μm/s. The simulation results for different adhesion settings are presented in Fig 10.

With an intermediate cell-cell adhesion and cell-basement membrane adhesion we see that the row of cells is predicted to buckle above the basement membrane (Fig 10a). A single-buckle develops at 40% compression. Theoretically buckling is the result of a local instability reflected by a bifurcation in the mathematical solution to the equations of static equilibrium (supplemented with appropriate constitutive equations). Traditionally the solutions to column and plate buckling are known as Euler buckling solutions. Higher buckling modes are obtained when the column or plate is suitably laterally restrained at various locations. Buckling of an epithelial layer has been theoretically studied previously and is relevant to morphogenesis and epithelial carcinogenesis [45–48].

**Fig 11:**
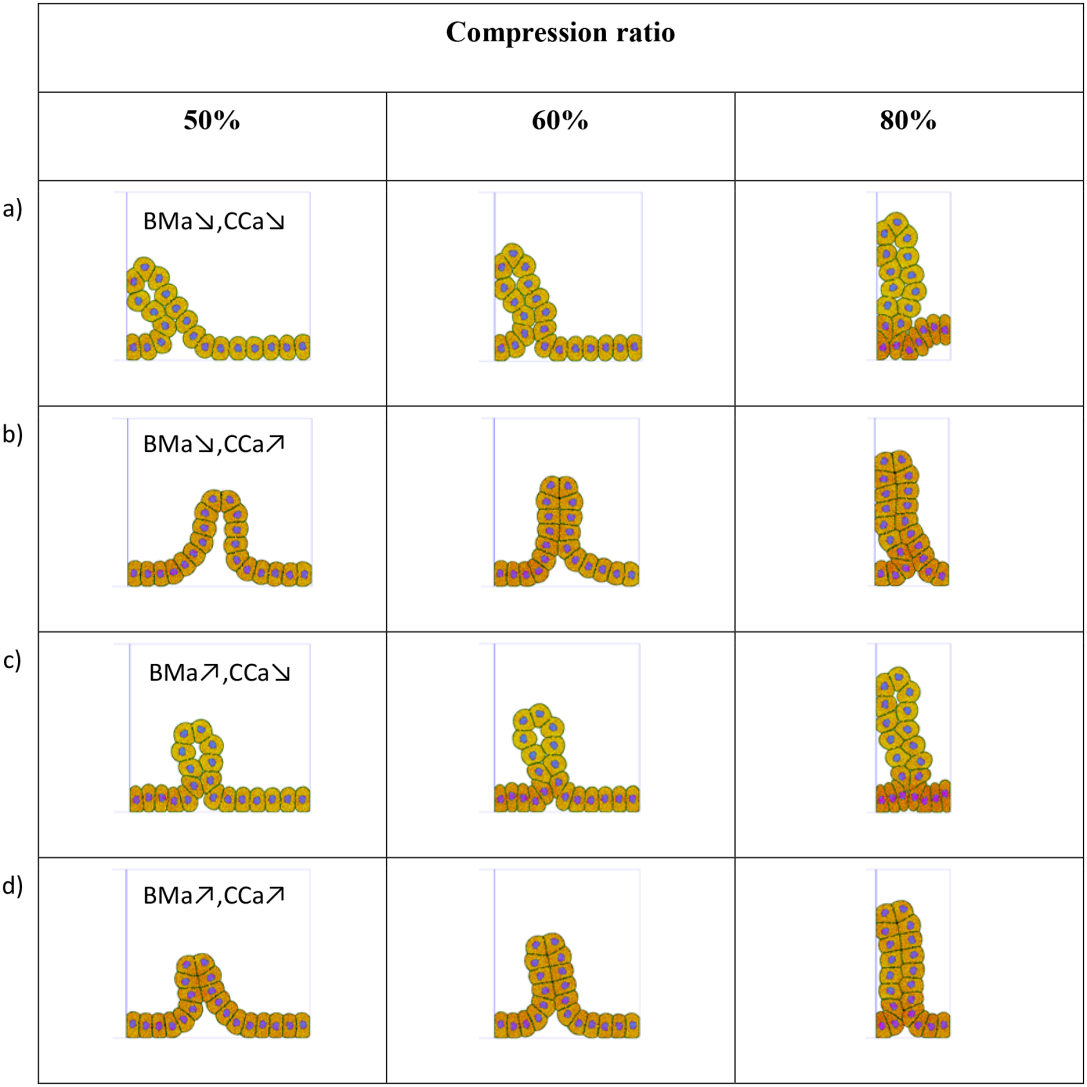
Influence of adhesion parameters on the shape of the buckle. a) Weak BM adhesion (BMa) and weak cell-cell adhesion (CCa) ($k_{BM}^{a}$ = 1×10^−11^ N/m, *δ*_*BM*_ = 0.1 μm, $k_{M}^{a}$ = 1x10^-11^ N/m, *δ*_*M*_ = 0.1 μm). b) Weak BM adhesion and strong cell-cell adhesion ($k_{BM}^{a}$ = 1×10^−11^ N/m, *δ*_*BM*_ = 0.1 μm, $k_{M}^{a}$ = 1x10^-9^ N/m, *δ*_*M*_ = 0.3 μm). c) Strong BM adhesion and weak cell-cell adhesion ($k_{BM}^{a}$ = 1×10^−9^ N/m, *δ*_*BM*_ = 0.3 μm, $k_{M}^{a}$ = 1x10^-11^ N/m, *δ*_*M*_ = 0.1 μm). d) Strong BM adhesion and strong cell-cell adhesion ($k_{BM}^{a}$ = 1×10^−9^ N/m, *δ*_*BM*_ = 0.3 μm, $k_{M}^{a}$ = 1x10^-9^ N/m, *δ*_*M*_ = 0.3 μm). The adhesion parameters influence both the shape of the cells and the deformation of the epithelial layer.

Although cells adhere to the basement membrane with a relatively weak (lateral) force, a progressive build up in longitudinal stress due to the compression (or cell growth) is relieved by the precipitation of a wrinkle or buckle. Cells within the buckle then detach from the basement membrane and move laterally. The shape of the buckle is determined by the ability of the cells to maintain a bending stiffness across the cell-cell contact, which is influenced by the membrane particles’ adhesion parameters (Fig 11).

With an increase in the attachment strength of the cells to the basement membrane, the sheet of cells is vertically restrained and buckling is suppressed (Fig 10b). No buckling is seen for strong BM adherence even at 40% compression. Instead the cells are squashed and observed to become ‘columnar’ in appearance (i.e. cells become tall and narrow, with elongated nuclei and a rounded top).

A decreased BM adhesion and cell-cell adhesion leads to bigger gaps in between cells at the basement where they rest. With the vertical restraint reduced, a higher buckling mode with 3 buckles develops at 40% compression (Fig 10c). This observed behaviour is qualitatively consistent with the Euler buckling theory.

We have also tested the influence of the number of cells and the drag factor on the buckling behaviour. Increasing the number of cells leads to the formation of higher buckling modes (the longer membrane tends to buckle at more points along its length). An increase in the drag factor leads to a shift of the buckle towards the moving wall, as motion does not propagate as fast from cell to cell due to the increased drag forces.

There have been several papers dedicated to modelling the buckling of epithelial layers. In [49] the authors used a continuum approach, creating a 2D model of an epithelial layer consisting of a beam connected to the underlying BM using viscoelastic springs. They studied the influence of different parameters (rate of growth, adhesion to BM) on the initiation of tissue buckling, but did not describe the shape or evolution of nonlinear post-buckled states. The approach was extended to the nonlinear regime in [46], combined with a study of the effect of growth-induced buckling of an epithelial layer on a thin substrate using a bilayer beam. When using the continuum approach, the buckling states of the system are determined from the minimisation of the system’s energy, and therefore a mathematical model of the system needs to be created; this makes the extension to 3D difficult. The approach cannot easily incorporate changes in the system (such as evolving contacts between cells and BM or between different cells in the epithelial layer), the parameters defining the system have little biological meaning (such as the bending stiffness of the beam), and does not provide any insight into how individual cells are affected by the buckling of the layer. Our modelling approach easily overcomes all these limitations.

Another approach in modelling epithelial layer deformation uses discrete, off-lattice cell-centre modelling approach [50]. In this approach cell centres are defined as nodes which evolve spatially according to an off-lattice definition of cell–cell mechanics. Spatial connectivity is determined by a Delaunay triangulation of cell centres, and the corresponding cell shapes are subsequently defined by the dual Voronoi tessellation. The interaction between the cells connected by the triangulation is modelled as linear springs. This model considers a deformable BM and includes its influence on the epithelial sheet deformation, but does not allow the two layers to separate. The model makes the same predictions regarding the influence of the cell-cell adhesion and cell-BM adhesion on the initiation of buckling and the shape of the compressed cells. The use of a triangulation to determine cell interactions means that new Delaunay triangulations need to be generated after cells change their position, therefore the approach does not scale well for large number of cells and in 3D. Cell shapes are only approximated by the Voronoi tessellation. While our model can perform all these tasks, it can also give a more accurate indication on what happens at cellular level.

**Fig 12:**
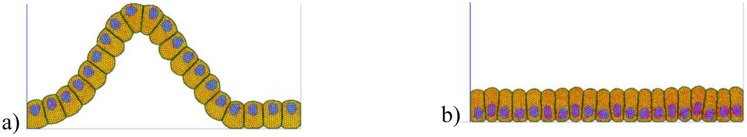
Influence of nuclei position on the behaviour of a cell layer under 40% compression. a) Elevated nuclei position facilitates buckling. b) Nuclei positioned near the BM suppresses buckling. The BM and cell-cell adhesion parameters are given by $k_{BM}^{a}$ = 1×10^−11^ N/m, *δ*_*BM*_ = 0.3 μm, $k_{M}^{a}$ = 5x10^-10^ N/m, *δ*_*M*_ = 0.2 μm. The position of the nucleus at the base of the cell reduces the likelihood of buckling.

To demonstrate the capability of our model to give insights on the effect of cell’s geometry on the behaviour of a biological system, a capability that is lacking in the previously used models, we will investigate the effect of cell polarisation on the buckling of an epithelial layer. In the colonic crypt epithelial cells are polarised. Following polarisation, it is observed that the nucleus sits at the bottom of the cell near the basement membrane [51]. It could be argued that polarisation has the advantage of facilitating secretory activity by the cell, that it may provide a greater signalling interaction to the underlying stroma, or it may protect the nucleus from environmental toxins generated by microorganisms in the luminal environment. But here we wish to test a fourth option, that there is a mechanical advantage to having a cell nucleus close to the basement membrane. We adjusted the initial nucleus height relative to the basement membrane (Fig 12) and performed a compression test on the cell layer. The results show that the buckling takes place much more easily for nuclei positioned higher away from the base, whereas buckling is delayed when nuclei are positioned nearer to the base. If a buckle does occur in the latter case, the buckle tends to be smaller and of lower amplitude. This suggests that in the colonic crypt the position of the nucleus at the base of the cell reduces the likelihood of buckling. Although not shown here, we have observed in our simulations that buckling tends to occur more readily when the nuclei positions are more variable relative to one another in an epithelial layer. It is noted that dysplastic cells tend to have more nuclear variability [52].

## Discussion

In this paper we present a framework for modelling biological tissues based on discrete particles. The cell and tissue models consist of a collection of particle types and a set of simple interactions between particles. The particles interactions are controlled by parameters define at particle level, which simplifies the implementation of the computational framework. An explicit solution method leads to an algorithm easy to implement on parallel hardware, such as GPU.

We have shown that an agent-based cell model with approx. 900 particles per cell can produce realistic cell morphologies that bear a remarkable resemblance to histological observations of cells *in situ*. By using just four particle types for each cell and defining a range of simple interactions between the particles types it is possible for our cell model to be calibrated to simulate mechanical loading experiments with great accuracy.

The cell model is able to reproduce key cell features observed in vitro and in vivo, such as cell rounding when the cell is suspended in a solution, cell attachment when a basement membrane is close by and variable spreading of the cell upon attachment to the basement membrane [34]. Consistent with experimental data, our cell model predicts an increase in cell stiffness when the cell is attached to a basement membrane, cell stiffness increasing with cell spreading [3].

The single cells can be assembled into sheets of cells with a variety of morphologies. We have studied the behaviour of a layer of cells under compression and shown that cell layer buckling behaviour can be significantly changed by modifying simple particle parameters.

Finally, we have shown that cell polarisation can reduce the likelihood of buckling of a sheet of cells. This raises the intriguing possibility that polarisation may not only be significant in signalling and secretory functions of cells, but also contribute to the mechanical stability of the cell sheet.

Cell behaviour and interactions between multiple cells or with the cell’s environment is complicated, involving many processes. There is a need to develop integrative tools to enable a system level understanding of these processes that are so critical to tissue development, homeostasis and disease. In this paper we have presented a discrete particle-based simulation framework and showed how, with relative simple adjustments to model parameters, we can reproduce cell mechanics, cell shape, histological aspects of epithelial geometries (e.g. columnar and squamous epithelia) and the onset of epithelial delamination from a basement membrane. Although we have primarily focussed on biomechanical aspects, we believe it is possible to incorporate chemical contributions to cell behaviour born from cell communication, as presented in [15]. Agent based fibrils can be easily incorporated in the framework [53]. Cell motility can be simulated by including tangential forces between the cell membrane and the BM particles. Cell proliferation and death can be incorporated in the framework, as well as sub-cellular particles which can move in the inter-cellular space or even diffuse through the cells. Therefore we consider this as a promising path to realistic, multiscale simulations of complex biological phenomena, with the potential to model a variety of mechanical and chemical interactions.
